# Parasite Prolyl Oligopeptidases and the Challenge of Designing Chemotherapeuticals for Chagas Disease, Leishmaniasis and African Trypanosomiasis

**DOI:** 10.2174/0929867311320250006

**Published:** 2013-08

**Authors:** I.M.D Bastos, F.N Motta, P Grellier, J.M Santana

**Affiliations:** 1 Pathogen-Host Interface Laboratory, Department of Cell Biology, The University of Brasília, Brasília, Brazil;; 2 Faculty of Ceilândia, The University of Brasília, Brasília, Brazil;; 3 UMR 7245 CNRS, National Museum Natural History, Paris, France

**Keywords:** Cell signaling, collagenase-like, protease, drugs, inhibitors, *leishmania*, oligopeptidase B, prolyl oligopeptidase, *trypanosoma brucei*, *trypanosoma cruzi.*

## Abstract

The trypanosomatids *Trypanosoma cruzi*, *Leishmania* spp. and *Trypanosoma brucei* spp. cause Chagas disease, leishmaniasis and human African trypanosomiasis, respectively. It is estimated that over 10 million people worldwide suffer from these neglected diseases, posing enormous social and economic problems in endemic areas. There are no vaccines to prevent these infections and chemotherapies are not adequate. This picture indicates that new chemotherapeutic agents must be developed to treat these illnesses. For this purpose, understanding the biology of the pathogenic trypanosomatid-host cell interface is fundamental for molecular and functional characterization of virulence factors that may be used as targets for the development of inhibitors to be used for effective chemotherapy. In this context, it is well known that proteases have crucial functions for both metabolism and infectivity of pathogens and are thus potential drug targets. In this regard, prolyl oligopeptidase and oligopeptidase B, both members of the S9 serine protease family, have been shown to play important roles in the interactions of pathogenic protozoa with their mammalian hosts and may thus be considered targets for drug design. This review aims to discuss structural and functional properties of these intriguing enzymes and their potential as targets for the development of drugs against Chagas disease, leishmaniasis and African trypanosomiasis.

## 1 INTRODUCTION

Severe diseases caused by pathogenic microorganisms affect millions of humans and animals around the world and have been a constant challenge. Among them, Chagas disease, leishmaniasis and human African trypanosomiasis (HAT) result from infections by the trypanosomatids* Trypanosoma cruzi*, *Leishmania *spp. and *Trypanosoma brucei *spp., respectively. These parasites are unicellular eukaryotic protozoa belonging to the order Kinetoplastida. In addition to their medical importance, trypanosomatids are excellent models for evolutionary studies because they have complex life cycles, have many different hosts, undergo cellular differentiation, possess unique structures and biochemical pathways and distinctive mRNA processing [1]. Different species of hematophagous insect vectors mediate trypanosomatid transmission to a wide range of vertebrate hosts, mainly in tropical and subtropical areas. It is estimated that more than 10 million people worldwide have Chagas disease, leishmaniasis or HAT with 600 million people being at risk to be infected in the poor areas where those illnesses are endemic, posing an immense social and economic burden [2]. There are no vaccines to prevent these diseases, and the drugs used to treat them are far from being satisfactory due to their lack of efficacy, toxicity, long treatment schedules, drug resistance and high cost.

Despite sharing many genomic, proteomic and cellular features, *T. cruzi*, *Leishmania* spp. and *T. brucei* spp. show unique parasite-host interactions and cause distinct diseases. Although emigration has made Chagas disease an international concern, its natural transmission cycle is found in Latin America where its vectors exist. Upon blood feeding, triatomine bugs transmit metacyclic trypomastigotes to mammals through contaminated feces. To infect tissues, *T. cruzi* trypomastigotes must cross vascular epithelia and the extracellular matrix before attaching to the surface of host cells. Once inside the cell, the parasite escapes from the lysosome and differentiates into a cytoplasmic amastigote that replicates and is transformed back into a trypomastigote before leaving the cell [3, 4]. Approximately 25% of infected patients can progressively develop inflammatory chronic Chagas disease that mostly affects the heart, esophagus and colon, leading to more than 10,000 deaths per year [2, 5].

After transmission through bites of sand fly vectors (frequently of genera *Phlebotomus* and *Lutzomya*) found in the Old and New worlds, infective *Leishmania* promastigotes are internalized by macrophages in which differentiation into amastigotes, which survive within vesicles, takes place. Leishmaniasis comprises a complex of diseases ranging from mild cutaneous to fatal visceral forms, both in humans and animals. Although the acute cutaneous disease can be controlled, life-long persistent reactivation of the infection causes severe post-kala-azar dermal leishmaniasis and opportunistic infections associated with the presence of HIV [6]. Leishmaniasis is endemic in more than 80 countries and causes at least 50,000 deaths per year worldwide [2].

The tsetse fly, *Glossina* spp., transmits African trypanosomes through its bite to humans and animals, causing African trypanosomiasis or sleeping sickness. Human infection with *T. b. gambiense* is responsible for over 90% of all reported cases and is found mainly in western and central Africa, whereas *T. b. rhodesiense* affects populations in the southern regions. Bloodstream forms of the parasite switch their variant surface glycoprotein, a major coat component, thus escaping from host immune response and ensuring persistent infection. HAT is the cause of more than 50,000 deaths annually [2].

This scenario requires the development of new molecules aiming at both prevention and control of these intriguing parasite infections. For this purpose, understanding the biology of trypanosomatid parasites, as well as their interactions with hosts, is a fundamental step in this direction. In this context, molecular and functional characterization of virulence factors is a good strategy to develop inhibitors that could be useful for effective chemotherapy. It has been well recognized that protease activities play crucial roles in both the physiology and infectivity of pathogens and are therefore considered potential targets for the development of new medicines.

The purpose of this article is to review the structural and functional properties of prolyl oligopeptidase and oligopeptidase B of *T. cruzi*, *Leishmania major* and *T. brucei* in parasite-host interactions and to discuss their potential as targets for selective inhibitors.

## 2 S9 SERINE PROTEASE FAMILY

**Fig. (1):**
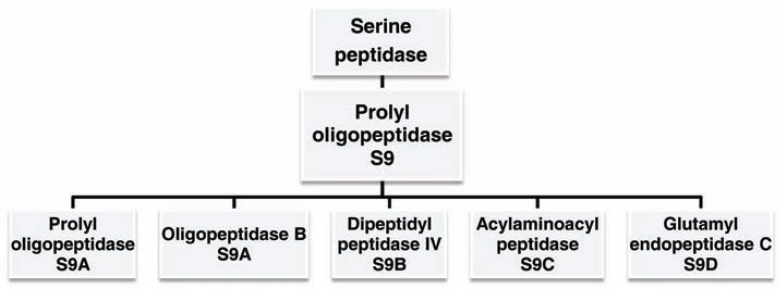
Schematic representation of S9 serine protease family.

Prolyl oligopeptidase is an S9 serine protease family consisting of the prototype prolyl oligopeptidase (POP, EC 3.4.21.26), oligopeptidase B (OPB, EC 3.4.21.83), dipeptidyl peptidase IV (DPPIV, EC 3.4.14.5), acylaminoacyl peptidase (ACPH, EC 3.4.19.1) and glutamyl endopeptidase C (GEP, EC 3.4.21.19) (Fig. **1**) [7]. In spite of these enzymes sharing a conserved three-dimensional structure, unlike POP and DPPIV, ACPH and OPB do not hydrolyze peptide bonds on the carboxyl side of proline residues. However, a common catalytic feature is their preference for cleaving peptides smaller than 3 kDa, such as many biological peptides, e.g., neurotransmitters and hormones. This feature has motivated several research groups to explore the physiological roles of human POP family members and the therapeutic potential of their inhibitors to treat neurological, hormonal and metabolic disorders such as Alzheimer’s disease, depression, abnormal blood pressure and type II diabetes. Two DPPIV inhibitors, vildagliptin and sitagliptin, are already in clinical use [8-12]. Due to their ability to cleave peptide bonds on the carboxyl end of proline residues, POP has also been studied as a potential therapeutic component for the treatment of celiac disease, a chronic enteropathy induced by immunotoxic and proline-rich gluten peptides [13]. Protein engineering based on mutagenesis has been developed to make POPs resistant to acid and digestive proteolysis, facilitating oral administration, and thus improving celiac disease therapy [14].

In addition to the physiological role of POPs in activation or inactivation of biological peptides, their orthologous enzymes in some pathogens have been described as virulence factors of infectious diseases, as in the case of trypanosomiasis and leishmaniasis.

## 3 OVERALL STRUCTURE AND CATALYTIC FEATURES

Structural data obtained by crystallization, three-dimensional modeling, molecular dynamics (MD), spectroscopic analysis and site-directed mutagenesis of POP family members of different species [15-24] have facilitated the understanding of how these structurally similar enzymes can present different biochemical features. In this section, we describe an overview of the structural properties of POP and OPB.

### 3.1 Prolyl Oligopeptidase (EC 3.4.21.26)

The crystallized structures of porcine [17], human [25], *Myxococcus xanthus* (MX PEP), *Sphingomonas capsulata* (SC PEP) [21] and *Aeromonas punctata *[22] POPs show a cylindrical architecture divided into an α/β hydrolase catalytic domain and a β-propeller domain. The α/β hydrolase is a structurally conserved domain with diverse catalytic functions also present in acetylcholinesterase, dienelactone hydrolase, lipase, thioesterase, serine carboxypeptidase, proline iminopeptidase and others (reviewed by [26]). The POP α/β hydrolase domain is formed by the folding of the C-terminal and a short extension of the N-terminal region. The three-dimensional model of *T. cruzi* POP (POPTc80) revealed that, as in the case of the porcine POP, the core of the α/β hydrolase domain contains eight helices surrounding the central β-sheets composed of eight strands, of which only one is antiparallel [27]. The POPTc80 active site consists of Ser548 as the nucleophile and His667 as the proton carrier, whereas Asp631 maintains the imidazole ring in a suitable position for capturing the serine proton during catalysis (Ser554, Asp641, and His680 in porcine POP) [17]. The catalytic Ser is part of the GGSNGG motif, which is highly conserved among POPs and is situated at the ‘nucleophilic elbow’ located at the interface between the α/β hydrolase and the non-catalytic β-propeller domain [27].

**Fig. (2):**
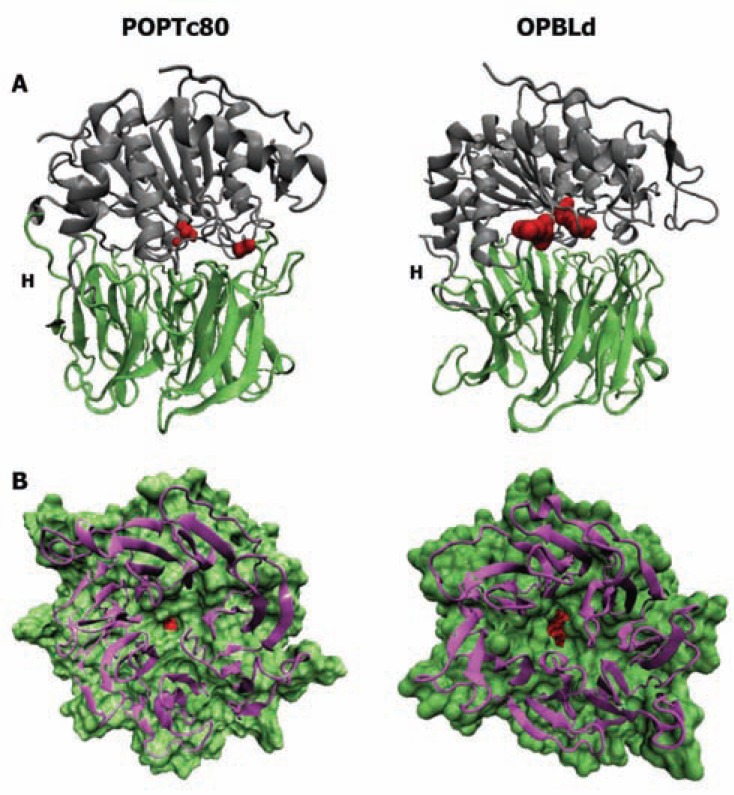
**Structures of POPTc80 and OPBLd.** (**A**) The catalytic α/β hydrolase domain (gray) is formed by the folding of the C-terminal and a short extension of the N-terminal regions. The non-catalytic β-propeller domain (green) is a symmetrical disk-like structure assembled by seven blades forming a central axis. H - hinge region. (**B**) A bottom view of the β-propeller domain pointing out the pore size. The catalytic site is shown in red. The POPTc80 model is based on the crystallographic structure of porcine POP. OPBLd PDB accession number: 2XE4.

The β-propeller domains are highly symmetrical disk-like structures assembled into radially positioned modules, also known as blades, around a central axis forming the central cavity (reviewed by [28]). This domain can consist of 4 to 10 blades in a large diversity of proteins that mediate different functions such as substrate/protein binding [29], transferase [30], lyase [31], signaling domain [32] and others [33, 34]. POP presents a seven-bladed β-propeller domain lacking the canonical closure [35] and hydrogen or disulfide bonds between the first and the seventh blade. From these two blades, two hinge-like strands that connect the β-propeller to the peptidase domain are formed. The internal cavity of the POP β-propeller domain forms a funnel with a lower face opening toward the external milieu and an upper face covalently joined to the α/β-hydrolase domain [17, 21, 22, 25] (Fig. **2A**).

Regardless of structure-based approaches, how the β-propeller domain contributes to POP activity and function has remained unclear. Due to the premise that POP cleaves only peptides up to 30 residues in length, it was initially proposed that the POP β-propeller would act as a gating filter by oscillating the blade movement, which would allow peptides, but not protein substrates, entry into the domain pore toward the active site. This proposal was based on engineering disulfide bonds between the first and the seventh blades, which resulted in the stabilization of the propeller domain and reduced enzymatic activity [18, 20]. However, further site-directed mutagenesis and MD showed that in spite of being an open Velcro domain, the POP β-propeller has a rigid and stable fold, making the substrate/inhibitor route through its tunnel improbable [19, 20]. In addition, the tunnel exit (4 Å in a resting state) is not wide enough to allow the passage of typical peptides (6–12 Å in diameter). On the other hand, a more recent MD and docking study suggested a role for the β-propeller domain in the egress of small products, instead of substrate/inhibitor entry into POP with a larger pore size [24]. The fluctuations in the β-propeller pore size among various species are due to the presence of lysine residues on the first and seventh blades (highly conserved in mammalian POPs) that hide the opening. POPs in which lysine residues are replaced by smaller amino acids can present a bigger pore size. However, replacement of lysine residues by long amino acids such as arginine (e.g., as seen in *Arabidopsis thaliana,* *T. brucei *and* T. cruzi *POPs) result in decreasing the pore size or completely hiding the pore opening [24] (Fig. **2B**).

Although there are still doubts concerning the mechanisms of the substrate/inhibitor entry and product egress, the opening between the two domains appears to be the potential substrate entry pathway. This new insight into the catalytic POP mechanism was provided by the crystallized structures of *S. capsulata* and *A. punctata *POPs in an open configuration, suggesting that the propeller domain acts as a lid, displaced by the two hinge linkers, to hide the active site [21, 22]. An insertion of a disulfide bridge between the two domains packed them together in a close structure and impaired the catalytic activity [20], reinforcing the concept that a physical step involving conformational change is the rate-limiting step, rather than chemical catalysis [36]. In addition, MD simulation and amino acid substitution also showed a loop (residues 193-209 in porcine POP) that detaches from the protein structure and may play a direct role in gating/recruiting substrates, as the channel through the loop region is both more flexible and wider, and thus, the more likely access pathway to the active site [23, 24].

**Fig. (3):**
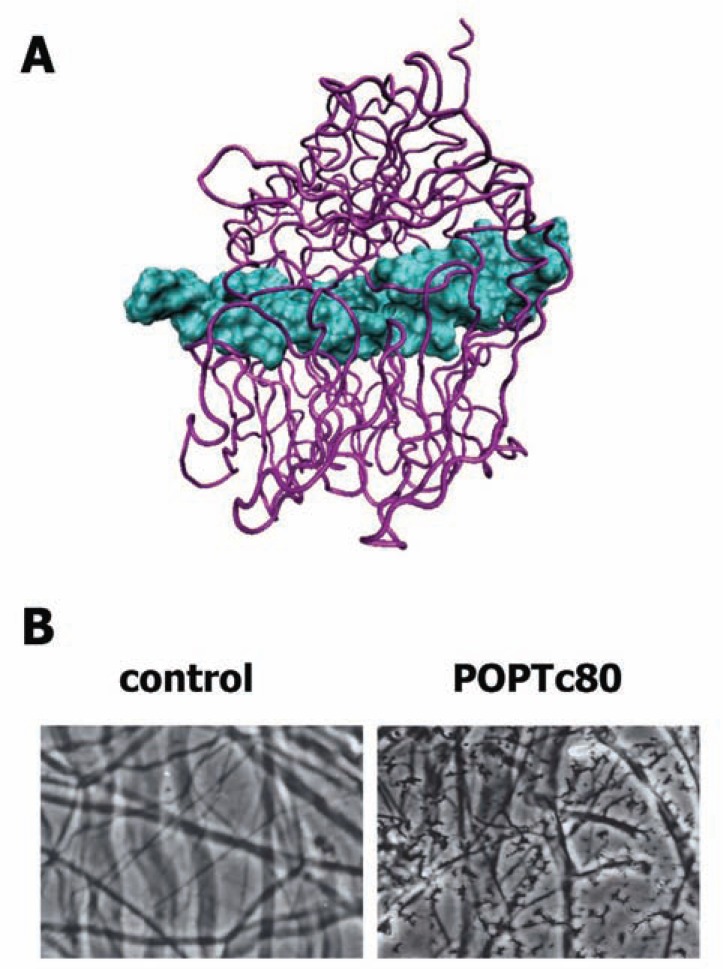
**Collagen hydrolysis by POPTc80.** (**A**) The docking of triple-helical collagen with POPTc80 shows that the collagen (blue) interacts in the vicinity of the α/β hydrolase and β-propeller domains (purple). (**B**) After 12 h of POPTc80 incubation with rat mesentery, hydrolysis and extensive degradation of collagen fibers are observed (JMS personal file).

Data from both experimental and computational studies have revealed that although POPs from different species show similar three-dimensional structures, differences among their primary sequences confer some divergent features such as conformational behavior, susceptibility to inhibitors and substrate specificity [23]. For instance, prolyl oligopeptidase of *T. brucei *(POPTb) and POPTc80 mediate hydrolysis of both denatured and native collagens, mainly type I [37, 38]. Molecular docking and dynamics between the triple-helical collagen structure and the proposed POPTc80 structural model suggest that collagen gains access to the active site via the interface region between the α/β-hydrolase and β-propeller domains facing the catalytic pocket [27] (Fig. **3A**). Most likely, collagen induces inter-domain movement of trypanosome POPs that corresponds to an induced fit mechanism. The root mean square deviation between the protein backbone atoms of the two complexes suggests that POPTc80 was able to accommodate such a large ligand without disrupting its structure. Site-directed mutagenesis, experimental enzymatic analysis and MD of POPTc80 validated this model, providing strong support for the hypothesis that the two domains can move in an open state that facilitates the entry and binding of large substrates to the catalytic site (our unpublished data).

Detailed studies on how larger substrates interact with some POPs for optimum activity may reveal not only unexplored structural features, but also unknown POP functions. Physiological roles of POPs are still a source of confusion, as *in vitro* activities and inhibition studies of these enzymes do not always correlate with *in vivo* observations (reviewed by [39]). Nevertheless, structure-based drug design strategies, in which theoretical predictions, chemical synthesis and biological experiments are performed in synergy, are important for discovering new drugs for the treatment of such diseases, such as the infectious diseases caused by trypanosomatids.

### 3.2 Oligopeptidase B (EC 3.4.21.83)

OPB is a processing enzyme with restricted substrate specificity that cleaves on the carboxy side of basic residues, with a preference for arginine over lysine and a further preference for cleavage after two adjacent pairs of basic residues and contains the conserved catalytic triad Ser, Asp and His [40, 41]. The first OPB (*Escherichia coli*) three-dimensional structural model was generated by homology modeling based on the high resolution X-ray structure of porcine POP [17, 42]. More recently, the crystallographic structure of *Leishmania donovani* OPB (OPBLd) was solved, confirming the archetypal assembly of the S9 peptidase family, a two-domain structure with the catalytic (peptidase) and the β-propeller domains connected by a hinge region [16, 21] (Fig. **2A**). The OPBLd catalytic domain is composed of N- and C-terminal regions that fold into a typical α/β hydrolase with a central eight-stranded β-sheet, eight α-helices and seven short sections of 3 helixes. The catalytic triad is hidden in a large cavity at the interface between the two domains, covered by the central tunnel of the β-propeller [16]. Similar to POP, the OPB β-propeller domain has a seven-fold repeat of four-stranded β-sheets radially arranged around a central tunnel, where the external opening is too small for substrate entry (Fig. **2B**). Although OPB does not present a canonical Velcro between the first and last blades of the β-propeller, hydrophobic interactions and a single hydrogen bond between residues Gly106 and Gln412 stabilize this domain [16].

Although OPB does not cleave after proline residues, it shares physicochemical and structural properties with POP family members [43]. Site-directed mutagenesis experiments suggest that the nine acidic residues conserved in OPB, but absent in POP, account for the preference for basic residues by OPB [44]. A pair of glutamic acid residues, Glu576 and Glu578, was identified in *Salmonella enterica *OPB (OPBSe), which may deﬁne P1 speciﬁcity and OPB catalytic activity toward the carboxy side of dibasic residues. A similar mutagenesis study showed that only Glu607 (corresponding to OPBSe Glu576) essentially defines the P1 specificity of *T. brucei *OPB (OPBTb) [45]. In addition, a critical role in the structural stability was proposed for a different glutamic acid residue in trypanosomes, the highly conserved Glu610. The single mutation of Glu610 to Gln markedly decreased OPBTb thermal and chemical stabilities. The structure of OPBLd also supports the importance of Glu621 (corresponding to OPBSe Glu576) in defining P1 specificity for basic residues and suggests that the Glu623 residue (corresponding to OPBSe Glu578) plays a role in holding the two domains in place for catalysis [16]. The residues that are supposed to be involved in POP substrate entry are not structurally conserved in OPB. Nevertheless, as for POP, the domain interface of OPB is important for both the functionality and substrate recognition properties of the enzyme [16, 23, 24].

Whether enzymatically active OPB is monomeric or assembles into an oligomeric state has been a controversial issue for the last two decades. An active dimer of *T. cruzi* OPB (OPBTc) was first suggested because its native form migrates as an 80-kDa protein when fully denatured and at an apparent molecular mass of 120 kDa upon electrophoresis without prior heating, which corresponds neither to a monomeric nor to a dimeric state [46]. In size exclusion chromatography (SEC) studies, OPBTc eluted as a 160-kDa dimer, despite its peculiar migration pattern in SDS–PAGE [47]. As for OPBTc, the dimerization of OPBTb had been proposed, based on SEC results, but was never proved [45]. It has been suggested that OPBTb does not form dimers, or any other type of multimers, in the absence or presence of reducing agents, which may be evident from its electrophoretic and chromatographic patterns [48]. This matter has not yet been evaluated for *Leishmania* OPBs, even upon the determination of the crystallographic structure of OPBLd. On the other hand, oligomeric assembly of OPBTc in an active stable dimer was recently demonstrated by analytical ultracentrifugation (AUC) [15]. Although chromatography is widely employed to estimate the apparent molecular mass of a protein, AUC is the method of choice for accurate molar mass determination and the study of self-association and heterogeneous interactions [49]. A deep analysis of OPBTc structural modifications accounting for the variations in its enzymatic activity under different conditions of pH, salt concentration and temperature showed that the OPBTc dimer is salt- and pH-resistant and devoid of intermolecular disulfide bonds [15]. These results brought new insights into the structural properties of OPBTc, contributing to future studies on the rational design of OPBTc inhibitors as a promising chemotherapeutic strategy for Chagas disease.

## 4 MOLECULAR MECHANISMS OF TRYPANOSOMATID POPS AND OPBS IN PATHOGENESIS

### 4.1 POPs

Chagas disease progression in an infected host depends on, among other factors, the ability of *T. cruzi* to spread in the blood, lymphatic system and tissues, where it is capable of infecting and replicating within many different cells [50]. This can reflect the diverse symptoms observed in the acute phase, such as fever, adenopathy, edema, hepatosplenomegaly, myocarditis and meningoencephalitis that can progress to “mega” syndrome in the heart, colon and esophagus found in the chronic phase of the disease (reviewed by [51]). The success of parasite dissemination is related to its capacity to migrate through the basement lamina and extracellular matrix (ECM) to gain access to the host cell surface. In this migration process, the parasite must interact with laminin, fibronectin, collagen, heparin and heparan sulfate and hydrolyze some of them [52]. As the major component, collagen forms the ECM scaffold and constitutes an important natural barrier against infections by pathogens [53]. 

POPTc80 has been described as a non-canonical collagenase for its ability to hydrolyze human type I and IV collagens [37], the major proteins of ECM and basement membrane, respectively. Moreover, POPTc80 also cleaves fibronectin [54], which is a proline-rich protein much like the collagens. However, its substrate specificity is restricted because purified POPTc80 does not cleave other large proteins such as albumin and laminin or even the small proteins such as insulin and cytochrome c [37]. The POPTc80 collagenolytic activity was demonstrated in a physiological context on rat mesentery, a tissue rich in type I collagen fibers, which is comparable to that mediated by the collagenase of *Clostridium histolyticum* [37] (Fig. **3B**). 

Some invasive microorganisms directly degrade collagens, which not only destroys the physical barrier but, in many cases, also induces host inflammatory responses that amplify the tissue damage and facilitate pathogen spreading [55]. Tissue injury induces the expression of several host matrix metalloproteases (MMPs) [56] that contribute to inflammation and tissue repair. For example, patients with acute *Entomoeba histolytica* colitis overexpress genes encoding MMP1 and MMP3 (secreted by fibroblasts) in colon biopsies [57]. Thus, it is suggested that these enzymes together with parasite cysteine proteases contribute to the extensive degradation of the collagen network observed during intestinal invasion, thus facilitating tissue invasion by *E. histolytica *[58]. Another consequence of collagen degradation is the generation of the tripeptide Pro-Gly-Pro, a ligand of CXCR1 and CXCR2 receptors that acts as a chemotactic, *in vivo* and *in vitro*, for leukocytes such as neutrophils [59, 60]. Evidence shows that Pro-Gly-Pro interaction with CXCRs occurs in a similar way to that of IL-8 via the conserved motif Gly-Pro [61] shared between them. In chronic neutrophilic inflammation, Pro-Gly-Pro is generated by POP activity on small collagen fragments pre-produced by MMPs [62]. In *T. cruzi*-infected heart tissue, increased levels of MMP2 and MMP9 were detected in association with leukocyte infiltration, leading to myocardial inflammatory response and significant tissue injury [63]. Treatment with the MMP inhibitor doxycycline significantly reduces severe myocarditis and increases survival rate, without any effect on parasitemia levels [63].

To play a role in the infectivity of mammalian cells, POPTc80 should be either on the surface or secreted by the parasite. A secretion assay showed increasing time-dependent POP activity, with the highest activity detected in the culture supernatants of trypomastigotes, followed by amastigotes, the infective and replicative forms of *T. cruzi*, respectively. The secreted activity is selectively inhibited by POPTc80 inhibitors [37]. In addition, differential expression of POPTc80 was further confirmed by western blots using specific antibodies against its recombinant form [27]. The use of an immunofluorescence assay to study POPTc80 cellular localization revealed that trypomastigote labeling is mainly associated with small vesicles surrounding the flagellar pocket, a specialized region of the plasma membrane involved in endocytosis and exocytosis in kinetoplastids. In contrast, amastigote labeling is detected in the entire cytoplasm and more concentrated in structures located at the opposite site of the kinetoplast/flagellar pocket area [54].

To evaluate the possible role of POPTc80 in infection by the parasite, several specific inhibitors have been developed and tested [54, 64-68]. It was observed that these molecules block host cell invasion by *T. cruzi* trypomastigotes in a dose-dependent manner with IC_50_ values ranging from 12 µM for the POPTc80 inhibitor, phenylpropylcarbonyl-L-Tic-pyrrolidine (see section 4), to 250 µM for *Z*-P-prolinal dimethylacetal, the prototype inhibitor of mammalian POPs. Reproducible results have been obtained with different *T. cruzi* strains Tulahuen, Y, Berenice, and 22 [54]. Preincubation of trypomastigotes with irreversible inhibitors of POPTc80 also blocks the invasion, indicating that the inhibitors act on parasite POP rather than on host cell POP. 

**Fig. (4):**
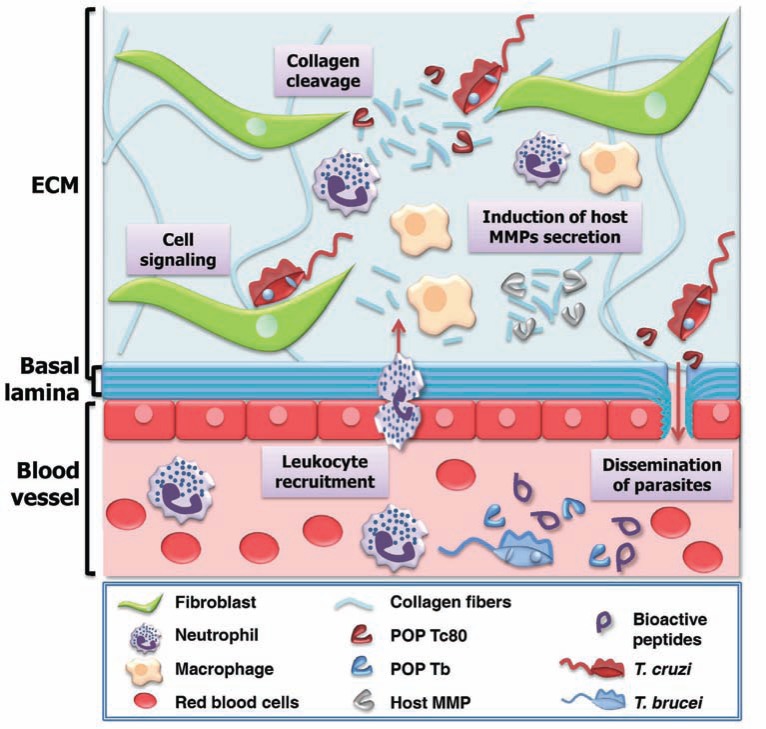
**Possible effects of trypanosome POP activity in host.** Collagen degradation by POPTc80 can facilitate *T. cruzi* migration through ECM. Activated immune cells release MMPs that amplify collagen degradation, and together with POP, generate chemotactic peptides, increasing leukocyte recruitment. POPTc80 can hydrolyze collagen present in basal lamina, contributing to trypomastigote dissemination in the blood and lymphatic system. POPTc80 can be involved in cell signaling that promotes *T. cruzi* invasion into host cells. POPTb released into the plasma by *T. brucei* can hydrolyze bioactive peptides and, depending upon the substrate, can act together with other parasite proteases such as OPB [89, 104]. Adapted from [115].

Cell invasion involves *T. cruzi* attachment to the host cells through interactions with ECM components and cell-surface molecules [69], which culminates in signal transduction events leading to the recruitment of host lysosomes at the parasite attachment location [70, 71]. The involvement of POPTc80 in cell invasion has been demonstrated by a parasite in-out immunostaining technique that allows differentiation of internalized parasites from those attached to host cells [70]. With increasing inhibitor concentration, a linear decrease of parasite number is observed in the host cells, coinciding with an increase in the number of attached parasites to the host cell surface. These results strongly suggest that POPTc80 inhibitors do not impair trypomastigote attachment but hinder host cell invasion itself [27, 54]. As attachment involves parasite binding to ECM components such as collagen [69], POPTc80 could facilitate invasion by cleaving the collagen that bridges parasite and host membrane proteins, allowing them to interact freely. In addition, modifications in the interactions among host integrins and collagens can trigger signaling cascades that could contribute to parasite entry, such as a modulation of the cytoskeleton [70]. Since POPs cleave a series of biologically active peptides [38, 72-74], POPTc80 could also contribute to the maturation/activation of parasite/host factors that trigger invasion (Fig. **4**).

Like POPTc80, *T. brucei *POP also mediates collagenolytic activity hydrolyzing purified type I human collagen and mesenteric stretched collagen fibers [38]. Such collagenolytic activity could contribute to parasite spreading in the host by facilitating its penetration through the endothelium of blood and lymphatic vessels. After the first hemolymphatic stage, HAT can progress to a second meningoencephalitic stage when parasites cross the blood–brain barrier (BBB) and invade the central nervous system [75]. The ability of *T. brucei* to cross the BBB seems to be driven by parasite proteases [76]*. T. brucei* cysteine proteases have been implicated in calcium-dependent signaling that promotes parasite transmigration [77, 78]. Host MMPs released during the inflammatory response against pathogen infection enhance collagen degradation in the BBB, a mechanism proposed in cerebral malaria [79], HAT [80], canine cerebral leishmaniasis [81] and Lyme disease [82]. In that way, POPTb could contribute to the disruption of the BBB by cleavage of collagen and/or its degradation products, leading to the production of Pro-Gly-Pro, a pro-inflammatory chemotactic peptide [60]. 

During infection, POPTb is detected, proportional to parasitemia rate, in the plasma of *T. brucei-*infected mice. It remains active up to the end of the parasitemia peaks, indicating that it is not inhibited by specific antibodies elicited during the infection [38]. Another pathogenesis mechanism associated with HAT involves the degradation of host circulating factors, such as hormones and neurotransmitter peptides by parasite proteases, and plays a role in neuroendocrine disturbances [83, 84]. Neurotensin, β-endorphin, bradykinin, GnRH and TRH are readily cleaved by POPTb after Ala and Pro residues present in these peptides [38]. Among them, abnormal levels of TRH and GnRH, both pituitary hormones at the top of the regulation cascade of peripheral hormones, seem to correlate with hypothyroidism and hypogonadism, frequent symptoms seen in HAT [85]. Pyroglutamyl peptidase (PGP; cleaves the p-Glu-His bond), which is also active in host plasma [86], might act in synergy with POPTb to decrease TRH and GnRH activities during parasitemia peaks or in untreated patients. POPTb could also hydrolyze other bioactive peptides that are known to be substrates of mammalian POPs, such as oxytocin, vasopressin, substance P, angiotensin, mastoparan and neuropeptide Y, as well as other Pro- and/or Ala-containing peptides [87, 88]. Thus, hydrolytic activity of POPTb may play a role in the ontogeny and/or maintenance of HAT (Fig. **4**).

### 4.2 OPB

OPB has been described as an important virulence factor in several trypanosome species [47, 89-91], and it is considered as a promising drug target as there are no OPB orthologs in mammals. Theoretically, this absence facilitates the development of selective drugs, presenting a lower risk for side effects.

**Fig. (5):**
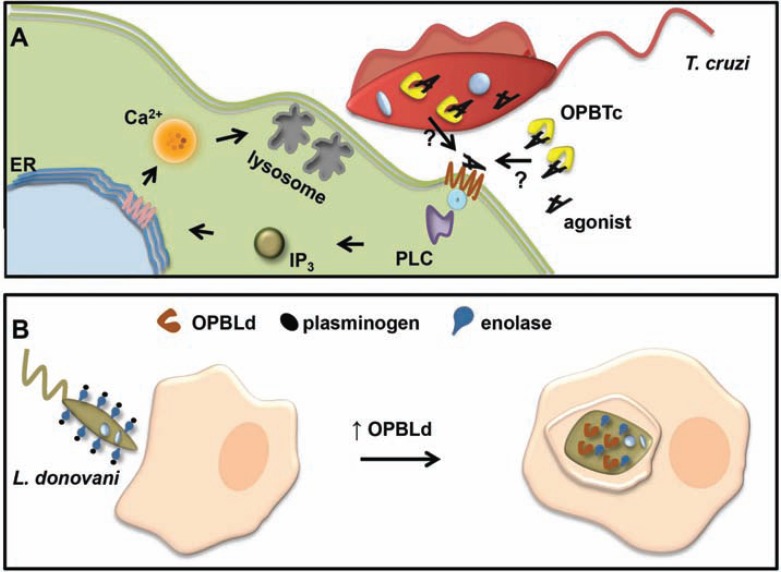
**Schematic representation of *T. cruzi* and *L. donovani* OPB role in host cell invasion.** (**A**) OPBTc cleaves an inactive precursor to generate an active Ca^2+^ agonist that binds to the host cell receptor, leading to the formation of inositol triphosphate (IP_3_) through the activation of phospholipase C and, consequently, to the mobilization of intracellular [Ca^2+^]. It is not yet clear if OPBTc interacts with its substrate inside or outside the parasite. ER, endoplasmic reticulum; PLC, phospholipase C; IP_3_, inositol triphosphate. Adapted from [50]. (**B**) During infection, *Leishmania* surface enolases bind to host plasminogen on the parasite cell surface. This interaction seems to help parasite entry into macrophages. As the parasite begins differentiating into amastigotes, OPB is up-regulated and acts on enolase and plasminogen clearance from the parasite surface, facilitating undetected replication of the amastigotes within the macrophage. Adapted from [107].

The physiological role of trypansomatid OPB was first investigated in *T. cruzi, *where it mediates pathogen invasion of non-phagocytic mammalian cells [47, 70, 71, 92]. To efficiently infect these cells, *T. cruzi *depends on two major lysosome-dependent pathways. One is a host plasma membrane-mediated invagination involving a kinase that generates an intracellular vacuole containing the parasite, which subsequently fuses with lysosomes [93]. The other process relies on a calcium-mediated signal triggered by trypomastigotes that recruit host cell lysosomes to the parasite attachment site where they are gradually fused with the plasma membrane, resulting in parasitophorus vacuole formation [70, 94]. Along with other well-characterized proteins such as cruzipain and metacyclic surface glycoprotein (gp82), OPBTc is a key component in the calcium-mediated signal transduction event that culminates in parasite host cell invasion [47, 71, 92, 95-97] (Fig. **5A**). OPBTc seems to trigger an unknown [Ca^2+^] agonist that binds to host cell receptors, leading to the formation of inositol triphosphate through the activation of phospholipase C and, consequently, to the mobilization of intracellular [Ca^2+^]. These [Ca^2+^] transients are responsible for the recruitment and fusion of host lysosomes at the *T. cruzi* trypomastigote invasion site. Unlike many pathogens that avoid fusion with lysosomes, *T. cruzi* depends on the presence of such an organelle to survive. The acidic environment provided by the lysosomes activates essential physiological processes, which lead to the differentiation of trypomastigotes into amastigotes [98], as well as to host immune system evasion [99, 100]. OPBTc [Ca^2+^]-signaling activity has been demonstrated in various experiments. First, the use of specific anti-OPBTc antibodies inhibited [Ca^2+^]-signaling in host cells [47]. To fully understand what role OPBTc plays in parasite physiology and host pathogenesis, *∆opb* null mutant *T. cruzi* has been generated. *In vivo* host cell invasion and infection establishment by these mutant parasites in mice were markedly reduced. Clearly, the invasion failure is associated with the inability of *∆opb*^(-/-) ^*T. cruzi* to mobilize host cell [Ca^2+^], which was demonstrated to be restored by exogenous OPBTc. Nonetheless, a vestigial [Ca^2+^]-signaling activity was observed in the infection by *∆opb*^(-/-)^ *T. cruzi*, indicating the importance and redundancy of this pathway in cell invasion by the pathogen [71]. However, the direct action of OPB in this process has not been established. It is yet to be determined whether the production of an agonist by OPBTc takes place in the cytoplasm or extracellular media. Sera or IgG purified from either infected or non-infected individuals enhances the enzymatic activity of OPBTc, which is secreted and may be associated with Chagas disease pathogenesis by its hydrolysis of host proteins [101].

Studies on *T. brucei *OPB (OPBTb) function during infection have also been addressed based on experiments using inhibitors, trypanocidal agents, neutralizing antibodies and, more recently, knockout experiments [48, 89, 102-104]. Interestingly, OPBTb is a target for pentamidine and suramin [103], drugs that are in use for the chemotherapy of African trypanosomiasis and whose mechanism of action is so far poorly understood [105]. OPBTb shows relevant *K_i_* values of 3.4 and 6.7 mM for pentamidine and suramin, respectively. However, there is no significant correlation between the inhibitory potency of OPBTb and its *in vitro* antitrypanosomal efficacy, suggesting that these inhibitors may be acting on multiple molecules [103]. As OPBTc is released into host circulation by dead and/or dying parasites where it remains fully active [89], it would be reasonable to associate OPBTb with abnormal peptide hormone metabolism – a typical symptom of sleeping sickness [84]. However, such a hypothesis could not be proved with the help of a knockout approach [104]. *∆opb*^(-/-) ^*T. brucei *is as virulent and pathogenic as the wild type parasites, considering that there are no significant differences in parasitemia or survival between mice infected with either type of the parasite. Another role of OPBTb was speculated to be its involvement in the crossing of endothelial barriers by the parasite [76], although *∆opb*^(-/-) ^*T. brucei *was shown to be as capable as the wild type in crossing the endothelial barriers. Nevertheless, *∆opb*^(-/-) ^*T. brucei *parasites show significantly elevated levels of POP activity that could indicate overlapping functions between OPBTb and POPTb. In the absence of OPBTb, POPTb or a closely related POP-like peptidase would hydrolyze physiological hormone substrates containing Arg/Lys or Pro and other substrates at a compensative turnover rate [38, 90] (Fig. **4**). Even with no explicit phenotype associated with *∆opb*^(-/-) ^*T. brucei*, it would not be prudent to rule out OPBTb as a virulence and pathogenic factor, considering that its *in vivo* functions can only be adequately studied in a ruminant host where a more natural chronic infection is achieved [104]. Additionally, OPB of *T. evansi*, an important veterinary pathogen that causes surra illness, hydrolyzes the peptide hormone atrial natriuretic factor (ANF), resulting in an increased blood volume, which is associated with lesions reported in the circulatory system during the disease [90].

*Leishmania *OPB function studies have more recently begun using gene knockout parasites [106, 107]. *∆opb*^(-/-) ^*L. major *parasites show a reduced ability to infect and proliferate within macrophages, suggesting that OPB may be associated with amastigote differentiation. However, there are no significant differences in the development of footpad lesions in BALB/c mice infected with wild type or *∆opb*^(-/-) ^*L. major *[106]. With respect to the knockout in *L. donovani*, a proteomics-based approach revealed that *∆opb*^(-/-)^ parasites accumulate enzymatically inactive enolase on their cell surface. Parasite enolase may bind host plasminogen, facilitating its entry into macrophages or preventing fibrin deposition on the extracellular parasite at the site of infection [107] (Fig. **5B**).

## 5 DEVELOPMENT OF DRUGS AGAINST TRYPANOSOME POPs AND OPBs

Parasitic and human POP active sites show structural divergences and inhibitors showing selectivity toward POPTc80 versus mammalian POPs might be expected. Several approaches have been undertaken for designing such inhibitors.

A peptide-mimetic approach has been developed based on inhibitors derived from the substrate recognition sequence (Leu-Gly-Pro), the C-terminal of which was modified with functional groups likely to interact with the protease active site: vinyl sulfone, 2-keto benzothiazole, nitrile or benzimidazole groups [108]. These compounds are reversible and competitive inhibitors. Compared to commercially available mammalian POP inhibitors (*Z*-Pro-L-prolinal dimethyl acetal, Boc-Asn-Phe-Pro-OH and Z-Pro-Pro-OH), a great improvement was observed in the inhibitory activity against POPTc80, with the nitrile derivative being the most effective inhibitor (Ki = 38 nM). These inhibitors show a high specificity toward POPTc80 when compared to other well-known *T. cruzi* proteases (OPB, Tc30 protease and cruzipain). They also inhibit *in vitro* invasion of a large range of mammalian cells by *T. cruzi* trypomastigotes [54]. However, selectivity toward POPTc80 versus rat POP was not achieved. Derivatives with the 3-acylisoxazole group and their corresponding isoxazolines were also evaluated [68]. The 3-acylisoxazole heterocycles undergo a ring-opening upon reaction with alcoholates giving rise to formylacetonitrile and the corresponding ester. The same reaction was expected in the POP active site, where the catalytic serine acts as a nucleophile. Z-Proline or N-(4-phenyl)butanoyl)proline was chosen for the P2-P3 position. Indeed, these derivatives were found to be potent inhibitors of POPTc80 and human POP activities with Ki values in the low nanomolar and picomolar range, respectively. A significant selectivity toward POPTc80 versus human POP was observed, with an index of selectivity of approximately 60 for the best one (the index is defined as Ki human POP/Ki POPTc80). Two promising compounds demonstrated *in vitro* inhibitory activity on the growth of different kinetoplastids known to possess POPs (*T. cruzi*, *L. donovani* and *T. b. rhodesiense*) with ED_50_s in a range of 2-13 µg/mL [38, 68]. One of them showed no cytotoxicity in KB cells. However, to the best of our knowledge, *in vivo* efficacy of these prolylisoxazole derivatives was not evaluated.

A combinatorial chemistry approach also resulted in the development of highly specific POPTc80 inhibitors. First, two orthogonal D-tripeptide combinatorial libraries composed of 15,625 structurally diversified tripeptides were assayed against POPTc80 activity. The importance of the Tic residue for inhibitory activity was demonstrated, and screening led to the discovery of a low micromolar inhibitor corresponding to H-Ipe-D-Tic-Glu(S-paratolyl)-OH [65]. Conformational analysis revealed a high degree of similarity in shape with a potent POP inhibitor, SUAM-1221 (phenylpropylcarbonyl-L-prolyl-pyrrolidine). Considering that the high hydrophobicity of the proline mimic Tic compared to proline could constitute a criterion of specificity between POPTc80 and mammalian POPs, a focused Tic-based library of 2,560 compounds was synthesized and screened against POPTc80 to study structure-activity relationships [64]. Only derivatives with pyrrolidine in the P1 position displayed an obvious inhibitory activity, an observation that is in good agreement with data reported in the literature for POP inhibitors, indicating that POPTc80 possesses a similar active site fold as other POPs, especially with regard to its S1 pocket. The stereochemistry of the Tic residue, the putative S2-binding moiety, was critical, with the L-Tic derivatives showing greater inhibitory potency. Several POPTc80 inhibitors, based on phenylpropylcarbonyl-L-Tic-pyrrolidine as the lead, were obtained with IC_50 _values in the very low nanomolar range. They are highly specific with no inhibitory activity toward *T. cruzi* OPB, Tc30 protease or cruzipain and inhibit the *in vitro* invasion of mammalian cells by *T. cruzi* trypomastigotes [54], as well as the *in vitro* growth of *T. brucei* bloodstream forms [38]. However, selectivity toward POPTc80 versus human POP was weak with a factor of selectivity of only 6.6 for the lead compound. To improve selectivity, structure-activity-relationships based on the lead compound were explored by introducing changes at the P1, P2 and P3 binding sites [67]. Improvement was achieved, but the index of selectivity of 80 was still rather moderate for the best inhibitor.

Although effective *in vitro*, the lead compound failed to demonstrate *in vivo* inhibitory activity in *T. cruzi*-infected mice (data not shown). The reasons of such an *in vivo* ineffectiveness are still unknown. Is POPTc80 crucial for the parasite survival in an *in vivo* physiological context or for a long term development of Chagas pathogenesis? Are there compensatory pathways that overcome POPTc80 inhibition *in vivo*? Is the pharmacokinetic of the lead compound appropriate to treat Chagas disease? Interestingly, this last question meets some recent remarks concerning POP inhibitors as therapeutic agents to treat cognitive deficit disorders [109, 110]. Although experimental data show that POP inhibitors have neuroprotective activities and the interest in POP inhibitors has been growing for a decade, few inhibitors have progressed into clinical trials. One reason that has been offered to explain this scenario is, with few exceptions, the lack of studies concerning bioavailability, pharmacodynamics, pharmacokinetics and toxicity of POP inhibitors, as well as the need of an optimal *in vivo* test model [109]. POPTc80 gene deletion is currently under investigation for evaluating the *in vivo* virulence potential of mutant parasites.

Peptidomimetic approaches were employed for designing POPTc80 inhibitors based on the reference pharmacophore prolyl-pyrolidine and the substrate recognition sequence Leu-Gly-Pro. The inhibitors obtained with such strategies were moderately selective although parasitic and human POP active sites show structural divergences. POP inhibitors studies are mainly based on the search of new peptidomimetics derived from the reference pharmacophore, but innovative chemical scaffolds based on non-peptide compounds and alkaloids are now emerging (reviewed by [109]). It may be expected that the access to compound libraries covering a large chemical diversity and to high-throughput facilities will be useful to look for new chemical scaffolds showing higher selectivity towards the parasite POP versus the human POP than the classical peptidomimetic approach undertaken until now.

Trypanosome OPBs have been identified as important virulence factors and are considered as promising drug targets, as no OPB orthologs were found in mammals. Interest was strengthened by the fact that *T. brucei* OPB appears to be one of the intracellular targets for a number of antitrypanosomal drugs (pentamidine, suramin, diminazene) [103]. Surprisingly, few studies have been performed to date to identify or design specific inhibitors. The biological role of OPB has been investigated using general serine peptidase inhibitors or trypsin-like peptidase inhibitors. OPBs are essentially arginyl hydrolases having Arg-Xaa as a major cleavage site (P1-P1’). The S1’ subsite seems to be an important determinant of substrate specificity, with a P1’ preference for Tyr, Ser, Thr and Gln [40]. Unfortunately, the P4-P1 specificity of OPBTb parallels that of many mammalian serine peptidases, which impedes the development of highly specific inhibitors [102]. Nevertheless, several classes of irreversible peptidase inhibitors containing basic amino acids in the P1 position (arginine, lysine or the arginine analog, 4-amidinophenylglycine) show inhibitory activity toward OPBTb and display moderate *in vitro* antitrypanosomal activity toward *T. brucei* bloodstream forms (EC_50_ of 27 µM for the best analog). Interestingly, the peptidyl aminoalkyl phosphonate diphenyl ester Cbz-Gly-(4-AmPhGly)^p^(OPh)_2_ showed *in vivo* antitrypanosomal properties in mice but unfortunately also exhibited toxicity [102]. In the search for specific inhibitors, it must be noted that protamines, which are basic arginine-rich peptides, might provide useful information for the design of specific OPB inhibitors. Indeed, they are potent nanomolar inhibitors with a 10^4^-fold specificity compared to trypsin [111]. It can be expected that, with the high-throughput screening approaches now used in academic laboratories and the development of chemical libraries containing a wide chemical variety of natural products, semi-synthetic or synthetic compounds, many new chemical scaffolds will emerge as promising OPB inhibitors with potential therapeutic applications, as seen for antimalarial drug development [112-114]. Development of selective OPB inhibitors will be facilitated by the recent crystal structure of OPBLm in complex with the protease inhibitor antipain [16].

## 6 CONCLUSION

Arresting infection by pathogenic trypanosomatids through the inhibition of a single enzyme will be a great scientific breakthrough because of the potential of designing new specific and efficacious drugs. However, life cycle features of *T. cruzi*, *Leishmania* spp. and *T. brucei *spp. make this goal a very difficult assignment. It is necessary to take into account that these parasites circulate in nature among many species of mammalian hosts, infect a wide range of cells and tissues and have sophisticated mechanisms to evade both innate and acquired immune responses. Thus, they must have many different molecular processes to successfully infect and live within hosts: a function accomplished by different proteins according to environmental conditions. Therefore, the rational design of drugs for Chagas disease, leishmaniasis and HAT must consider several enzymes from different physiological pathways as targets. In this context, a drug cocktail against diverse targets is the best strategy to efficiently treat these infections. Such drug combinations will also have the advantage of delaying the emergence of drug resistance. Among the molecules that have already been identified as virulence factors in parasitic protozoa, oligopeptidase B and prolyl oligopeptidase can be considered as potential target candidates for drug development.
